# Distribution of *de novo* Donor-Specific Antibody Subclasses Quantified by Mass Spectrometry: High IgG3 Proportion Is Associated With Antibody-Mediated Rejection Occurrence and Severity

**DOI:** 10.3389/fimmu.2020.00919

**Published:** 2020-06-02

**Authors:** Vincent Pernin, Anais Beyze, Ilan Szwarc, Nicole Bec, Céline Salsac, Esther Perez-Garcia, Georges Mourad, Pierre Merville, Jonathan Visentin, Helene Perrochia, Christian Larroque, Lionel Couzi, Moglie Le Quintrec

**Affiliations:** ^1^Department of Nephrology, Dialysis and Transplantation, Montpellier University Hospital, University of Montpellier, Montpellier, France; ^2^Institute for Regenerative Medicine & Biotherapy (IRMB), University of Montpellier, INSERM, Montpellier, France; ^3^Department of Nephrology, Transplantation, Dialysis and Apheresis, Pellegrin University Hospital, Bordeaux, France; ^4^Immuno ConcEpT, UMR CNRS 5164, Bordeaux, France; ^5^Université de Bordeaux, Bordeaux, France; ^6^Department of Immunology and Immunogenetics, Pellegrin University Hospital, Bordeaux, France; ^7^Department of Pathology, Montpellier University Hospital, Montpellier, France; ^8^Institut de Recherche en Cancérologie de Montpellier (IRCM), INSERM U1194, Montpellier University, Institut Régional du Cancer de Montpellier (ICM), Montpellier, France

**Keywords:** *de novo* DSA, IgG subclass, antibody-mediated rejection, kidney transplantation, mass spectrometry

## Abstract

Donor-specific antibodies (DSAs) are the main risk factor for antibody-mediated rejection (ABMR) and graft loss but could have variable pathogenicity according to their IgG subclass composition. Luminex-based test might lack sensitivity for the detection of IgG subclasses and this test does not allow quantifying the relative abundance of each IgG subclass. We investigated the precise repartition of each DSA subclass and their role in ABMR occurrence and severity, using an innovative mass spectrometry-based method. Between 2014 and 2018, we enrolled 69 patients who developed *de novo* DSA (*n* = 29 without ABMR, and *n* = 40 with ABMR) in two transplant centers. All IgG subclasses were detected in every samples tested: 62.7% were IgG1, 26.6% were IgG2, 6.6% were IgG3, and 4.2% were IgG4. The IgG3 proportion was significantly higher in the ABMR+ compared to the ABMR– group (8.4% vs. 5.6%, *p* = 0.003). The proportion of IgG1, IgG2, and IgG4 of DSA was similar between the two groups. Higher IgG3 level was associated with higher C4d deposition, higher microvascular inflammation scores, and glomerular filtration rate decline >25%. IgG3 proportion was not correlated with DSA MFI. Multivariate analysis showed that proteinuria and high level of IgG3 DSA were the only two factors independently associated with ABMR. In conclusion, *de novo* DSA are always composed of the four IgG subclasses, but in different proportions. High IgG3 proportion is associated with ABMR occurrence and severity and with poorer outcome, independently of DSA MFI.

## Introduction

Antibody-mediated rejection (ABMR) is now recognized as the leading cause of long-term renal transplant loss (1). ABMR results from the interaction between endothelial cells and donor-specific antibodies (DSAs), mainly against HLA antigens, leading to endothelial cell activation, complement activation via the classical pathway, inflammatory cell recruitment within the graft microcirculation (glomerular capillaries and peri-tubular capillaries), and graft dysfunction (2).

*De novo* DSA (i.e., DSA appearing after transplantation) are detected in ~20% of transplant recipients in the first 5 years (3), and are a major risk factor for ABMR and graft loss. However, the clinical course after *de novo* DSA detection is very heterogeneous, ranging from absence of detectable graft injury to rapid graft function deterioration and graft loss (4). Therefore, anti-HLA antibodies seem to have variable pathogenicity. DSA level (mean fluorescence intensity, MFI, using the Luminex Single Antigen test) and ability to bind to the complement component 1q (C1q) contribute to the graft rejection risk, but do not explain the outcome disparities (5, 6).

On the other hand, the different immunoglobulin (Ig) G subclasses significantly modulate antibody function and could be crucial for DSA pathogenicity. Indeed, each IgG subclass contributes differently to complement-dependent cytotoxicity (CDC) and antibody-dependent cellular cytotoxicity (ADCC). IgG3 displays the greatest potential for complement activation, followed by IgG1 (7). IgG2 and IgG4 show little or no binding to C1q and complement activation. Moreover, IgG3 and IgG1 have the best affinity for the FcγRIIIa activating receptor for natural killer cell-mediated ADCC (8).

Several research groups had studied the DSA subclass distribution with the Luminex test, and some of them found a correlation between IgG3 detection and poor outcome after renal transplantation (9, 10). However, this test might lack sensibility for IgG subclass detection, as suggested by the fact that any subclass was detected in only about of 20% of iDSA analyzed in these studies. Furthermore, the Luminex test does not allow quantifying the relative abundance of each IgG subclass.

Therefore, we developed an innovative mass spectrometry-based method to assess the relative IgG subclass composition of DSA after their capture on HLA Luminex beads. The aim of this study was to evaluate the distribution of the different DSA subclasses and their role in ABMR occurrence and severity.

## Materials and Methods

### Study Population

**Figure 1:**
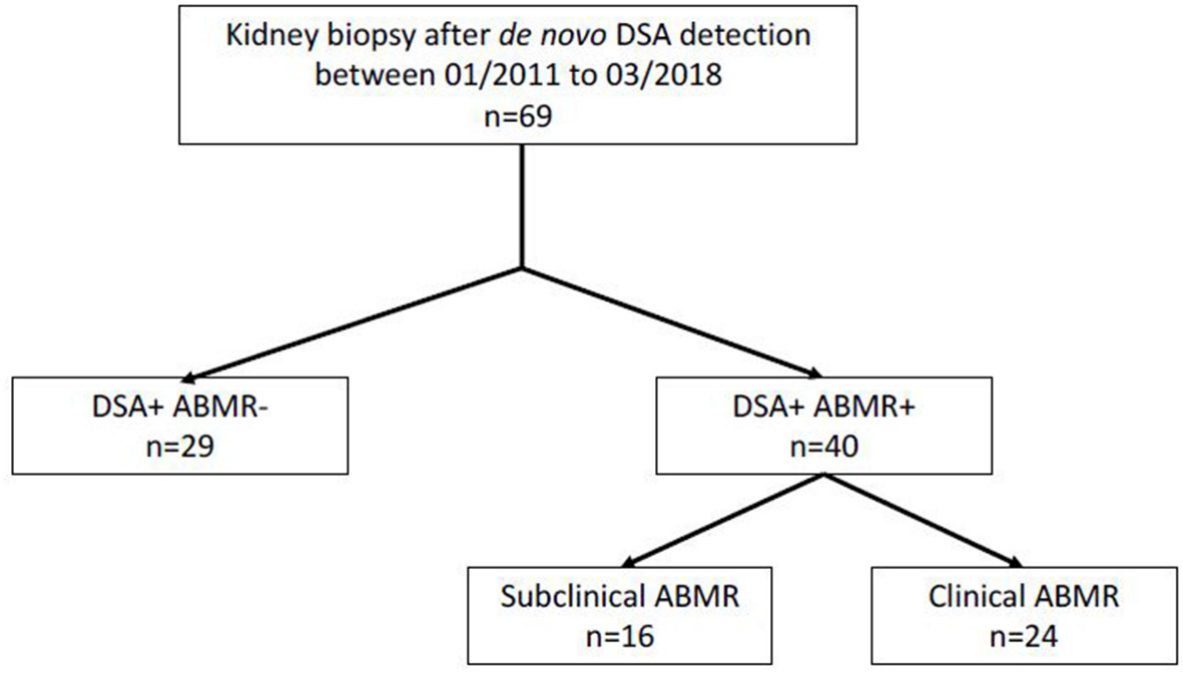
Study flow chart.

From 01/01/2014 to 01/03/2018, all patients who developed *de novo* DSA and had a kidney biopsy after kidney transplantation were prospectively enrolled at two French transplant centers (Montpellier Hospital and Bordeaux Hospital) (Figure 1). At both centers, routine anti-HLA DSA screening with a single antigen bead (SAB) assay (One Lambda, Canoga Park, CA) was performed at day 0, and then at month 3, month 12, and every year after transplant, and in the case of increased creatinine or proteinuria. All serum samples were pre-treated with EDTA to avoid the prozone effect (11, 12), and beads with a normalized MFI value >1,000 were considered positive. *De novo* DSA was defined as an antibody that was detected only after transplantation. For patients with multiple DSA, only the immunodominant DSA (iDSA), defined as the DSA with the highest MFI value, was considered for the subclass distribution analysis. Kidney biopsy was performed at the time of DSA detection, or of increased creatinine or proteinuria. Serum samples were prospectively collected for DSA subclass analysis at the time of kidney biopsy, and stored at −80°C. Patients with combined organ transplantation, ABO-incompatible kidney transplantation, and patients with DSA or positive CDC cross-match were excluded. The recipients' characteristics [age, sex, transplant number, blood type, initial nephropathy, immunosuppression, T cell-mediated rejection (TCMR) prior to DSA occurrence], kidney function at biopsy time, and the donors' characteristics were collected by reviewing the medical records. All enrolled patients gave their written informed consent for using their data for research purposes according to the Declaration of Helsinki (clinicaltrials.gov identifier: NCT04026087).

ABMR was diagnosed according to the last diagnostic criteria revised at the 2017 Banff Conference (13). C4d staining was assessed by immunochemistry (rabbit monoclonal anti-C4d antibody, clone A24-T, DB Biotec). Clinical AMBR was defined by an estimated glomerular filtration rate (eGFR) decline >20% (calculated with the MDRD formula) (14) or occurrence of proteinuria (proteinuria/creatininuria ratio >0.5 g/g) at the time of graft biopsy associated with ABMR histological criteria [C4d staining score; glomerulonephritis + peritubular capillaritis (g+ptc) score; intimal or transmural arteritis (v) score; tubulitis + interstitial inflammation (t+i) score; chronic glomerulopathy (cg) score; mesangial matrix expansion (mm) score, vascular fibrous intimal thickening (cv) score; interstitial fibrosis and tubular atrophy (IFTA) score]. Subclinical ABMR was defined by the presence of DSA and ABMR histological criteria, but without kidney dysfunction.

### Characterization of Immunodominant DSA Subclasses

#### Isolation of Immunodominant DSA

##### Protein G affinity purification

Total IgG was purified from 500 μl of plasma by protein G-based affinity chromatography using the Nab Protein G Spin Kit (Thermofisher) according to the manufacturer's instructions. Samples were dialyzed with phosphate-buffered saline and concentrated using Amicon centrifugal filters (100 kDa cutoff) to a final IgG concentration of 10 mg/ml. IgG concentration was determined by measuring the absorbance at 280 nm on a Nanodrop spectrophotometer.

##### DSA IgG isolation using monospecific HLA luminex beads

DSA were isolated from total IgG using Luminex FlowPRA® single-antigen beads (One Lamda, Inc.). These beads, which are routinely used for DSA detection, are coated with a single HLA antigen. Each bead batch contain beads specific for nine different HLA antigens. First, beads that interacted with the DSA were sorted by flow cytometry (Facs ARIA, Becton Dickinson). Then, total IgG were incubated with the selected beads for 1 h, and beads were washed three times with ammonium bicarbonate buffer.

In parallel, the specific binding of DSA to HLA Luminex beads was controlled by flow cytometry, according to the manufacturer's instructions.

#### Quantification of the iDSA Subclasses

##### Sample preparation for mass spectrometry

Total IgG (10 μg) and DSA-specific IgG were resuspended in 200 mM Tris buffer, pH 8.6, with 8 M urea for denaturation. After cysteine reduction (incubation with 10 mM DTT and 100 mM NH_4_HCO_3_ at 55°C for 45 min) and protein alkylation (incubation with 55 mM iodoacetamide and 100 mM NH_4_HCO_3_ in the dark at room temperature for 30 min), samples were diluted four times with protease buffer. Trypsin (sequencing grade, Promega) was diluted in 100 mM NH_4_HCO_3_/5 mM CaCl_2_, to a final protease-to-substrate ratio of 1:20, and samples were digested at 37°C overnight. Trifluoroacetic acid concentration was adjusted to 0.05% in samples prior to desalting using Omix C18 tips (Agilent Technologies). The resulting peptides were dried under vacuum and stored at −20°C until analysis.

##### LC separation and MS/MS detection

The peptide mixtures were dissolved in 10 μl of 0.1% formic acid (FA), and loaded on an Ekspert 425 nanoLC system (Eksigent) equipped with a C18 column (Discovery BIO Wide Pore, Supelco). The mobile phases were solvent A (water, 0.1% FA) and B (acetonitrile, 0.1% FA). Injection was performed with 98% solvent A at a flow rate of 5 μl/min. Peptides were separated at 30°C with the following gradient: 2% to 40% B for 105 min, 40–80% B for 5 min. The column was washed with 80% solvent B for 5 min and equilibrated with 98% solvent A. Peptide separation was monitored online with the coupled TripleTOF 5600 mass spectrometer (Sciex). External calibration was performed using trypsin-digested beta-galactosidase (Sciex). The total ion chromatogram acquisition was made in information-dependent acquisition (IDA) mode using the Analyst TF v.1.7 software (Sciex). Positive ion profiling was performed from m/z 350–1,500, followed by a MS/MS product ion scan from m/z 100–1,500 with the abundance threshold set at more than 100 cps. The accumulation time for ions was set at 250 ms for MS scans, and 100 ms for MS/MS scans. Target ions were excluded from the scan for 10 s after detection. The IDA advanced “rolling collision energy (CE)” option was employed to automatically ramp up the CE value in the collision cell as the m/z value was increased. A maximum of 25 spectra were collected from candidate ions per cycle.

##### IgG sub-class relative quantification

Peptides and proteins were identified using the ProteinPilot software v.5.0 (Sciex). Each MS2 spectrum was searched against the UniProt/Swiss-Prot database using the following parameters: sample type: identification; cys alkylation: iodoacetamide; digestion: trypsin; instrument: TripleTOF 5600; special factors: urea denaturation; species: *Homo sapiens*; search effort: thorough; ID focus: biological modifications.

Among the available label-free quantification strategies, spectral counting, defined as the number of peptides assigned to a protein in an MS2 experiment, is a straightforward quantification method especially suitable for estimation of high abundance proteins (15).

Each IgG subclass was quantified using two unique proteotypic peptide sequences listed in Supplementary Table 1. Some of these sequences were previously selected for IgG subclass quantification studies by LC-MS in multiple reaction monitoring mode (16–18). Only proteotypic peptides identified with a confidence score ≥99 were retained. For each IgG subclass, the spectral count was determined. As the sequence SCDTPPPCPR is present, three times in the IgG3 heavy-chain backbone, counts for this sequence were divided by 3. To evaluate the relative abundance of each IgG subclass in a sample, spectral counts were normalized to the total sum of the spectral counts of all subclasses.

This choice was validated by the similar results obtained in two test samples using the recognized immunoturbidimetric method (Biomnis, Lyon, France) (data not shown).

### Statistical Analysis

Normality was assessed with the Shapiro-Wilk test and graphically using quantile–quantile plots. Continuous variables were described as means (±SD) or medians [25th and 75th percentile] according to their distribution. Categorical variables were represented as proportions. The *t*-test, Wilcoxon test, chi-square, and Fisher's exact tests were used to compare groups, as appropriate. Receiver operating characteristic (ROC) curves were used to identify the IgG3 DSA and MFI cutoff values that best predicted ABMR, and the best possible cutoff was defined with the Youden Index ([sensitivity + specificity] – 1).

The relationship between ABMR and the different clinical and biological variables of interest was assessed by logistic regression analysis. The included variables were as follows: patient characteristics (sex, age, and transplant number), donor and graft characteristics (sex, age, donor origin, cold ischemia time, number of HLA mismatches, and delayed graft function), immunosuppressive treatment (induction therapy and therapy at biopsy time), eGFR, proteinuria/creatininuria ratio, DSA characteristics (number of DSA, sum of DSA MFI, iDSA MFI, and IgG3 DSA level: high vs. low). The relevant variables for ABMR prediction were identified by multivariable analysis with a stepwise backward elimination. Variables with a *p* < 0.2 were kept in the model.

GFR decline was defined by an eGFR decrease >25% or need of hemodialysis (graft loss). The eGFR decline-free survival rate was assessed with the Kaplan–Meier method, and compared among groups with the log-rank test. The eGFR decline-free survival rate was calculated from the date of kidney biopsy to the date of eGFR decrease or graft loss. Cox proportional-hazards models were used to estimate the hazard ratio (HR) and 95% confidence intervals (CI) for each studied variable: the previously described patient characteristics and immunological factors and also the histological characteristics according to the Banff 2017 criteria. Factors with a *p* < 0.20 were included in the multivariate Cox model.

*p* < 0.05 were considered significant. Analyses were performed using GraphPad Prism version 8.00 (GraphPad Software, La Jolla, CA, USA) and STATA version 12 (College Station, TX: StataCorp LP).

## Results

### Patients' Characteristics

From January 2014 to March 2018, *de novo* DSA antibodies were detected in 69 patients who underwent kidney biopsy in the two transplant centers. Forty patients had DSA with ABMR at biopsy time (DSA+/ABMR+ group), and 29 patients had DSA without ABMR (DSA+/ABMR– group) (Figure 1). Overall, the median interval between transplantation and DSA detection was 5.3 years and was longer in the DSA+/ABMR+ than in the DSA+/ABMR– group (7.5 vs. 4.0 years; *p* = 0.01). Similarly, immunosuppression at biopsy time, cold ischemic time, and proteinuria/creatininuria ratio were different in the two groups (Table 1). Patients in the DSA+/ABMR+ group took more frequently cyclosporine, had longer cold ischemic time (19.7 vs. 14.4 h, *p* = 0.01), and higher proteinuria/creatininuria ratio (0.36 vs. 0.19 g/g, *p* = 0.03). Conversely, eGFR at biopsy time was similar between groups.

**Table 1:** Patient characteristics at biopsy time.

	**All patients (*n* = 69)**	**DSA+ ABMR–(*n* = 29)**	**DSA+ ABMR+ (*n* = 40)**	***p*-value**
**Recipient characteristics**				
Age (years), mean ± SD	47.3 ± 16.7	46.8 ± 14.8	47.6 ± 18	0.82
Male sex, *n* (%)	39 (56.5)	17 (58.6)	22 (55.0)	0.81
Transplant number, first Tx (%)	58 (84.1)	26 (89.7)	32 (80)	0.33
Initial nephropathy, *n* (%)				0.10
Diabetes	7 (10.1)	4 (13.8)	3 (7.5)	
Glomerulopathy	24 (34.8)	10 (34.5)	14 (35)	
PKR and uropathy	20 (29.0)	4 (13.8)	16 (40)	
Others	18 (26.1)	11 (37.9)	7 (17.5)	
**Donor characteristics**				
Age (years), mean ± SD	42.7 ± 17.4	46.2 ± 16.6	40.2 ± 17.8	0.16
Deceased donors, *n* (%)	61 (88.4)	25 (86.2)	36 (90)	0.71
**Transplant characteristics**				
Cold ischemic time	17.6 ± 8.6	14.4 ± 7.1	19.7 ± 8.9	0.01
Delay graft function	5 (6.3)	3 (10.3)	2 (5)	0.64
Induction therapy				0.79
ATG, *n* (%)	48 (69.6)	21 (72.4)	27 (67.5)	
Basiliximab, *n* (%)	21 (30.4)	8 (27.6)	13 (32.5)	
HLA mismatch (ABDR), mean ± SD	4.4 ± 1.4	4.4 ± 1.3	4.1 ± 1.5	0.35
**Characteristics of all DSA:**				
Number (mean ± SD)	1.31 ± 0.70	1.24 ± 0.64	1.37 ± 0.74	0.44
HLA class (class I, class II, both), *n*	16/48/5	6/22/1	10/26/4	0.56
**Characteristics of iDSA**				
HLA class: class I/class II, *n* (%)	18 (26.1)/51 (73.9)	7 (24.1)/22 (75.9)	11 (27.5)/29 (72.5)	0.79
MFI median (IQR)	6,500 [3,200–19,000]	5,460 [1,975–8,650]	7,700 [4,325–20,000]	0.05
Time post Tx (years), median [IQR]	5.3 [1.5–9.3]	4.0 [0.6–6.6]	7.5 [2.3–10.8]	0.01
**Immunosuppression**, ***n*** **(%)**				
Tacrolimus	41 (59.4)	23 (79.3)	18 (45)	0.01
Cyclosporine	13 (18.8)	1 (3.4)	12 (30)	0.01
mTORi	12 (17.4)	5 (17.2)	7 (17.5)	0.99
IMPHDi ou AZA	62 (89.9)	26 (89.7)	36 (90)	0.99
Corticoids	50 (72.5)	22 (75.9)	28 (70)	0.78
Serum creatinine (μmol/L)	146 [113–189]	145 [106–188]	148 [114–191]	0.64
eGFR (MDRD) (ml/min/1.73 m^2^)	46.0 [31–57.5]	45 [30–63]	47 [32–55.7]	0.69
Proteinuria/creatininuria ratio (g/g)	0.3 [0.14–0.77]	0.19 [0.09–0.62]	0.36 [0.18–0.90]	0.03
**Rejection treatment**				
Plasma exchange + high dose IVIg	38 (55.1)	0 (0)	38 (95)	<0.001
Rituximab	12 (17.4)	0 (0)	12 (30)	<0.001
Follow-up post biopsy (years)	1.7 ± 1.2	1.6 ± 1.2	1.7 ± 1.2	0.69
**Histological characteristics**				
g score, mean ± SD	1.31 ± 1.23	0.18 ± 0.48	2.13 ± 0.92	<0.001
cpt score, mean ± SD	1.16 ± 1.04	0.52 ± 0.83	1.63 ± 0.93	<0.001
i + t score, mean ± SD	1.43 ± 1.67	1.07 ± 1.71	1.69 ± 1.61	0.018
cg score, mean ± SD	0.34 ± 0.73	0.07 ± 0.25	0.55 ± 0.89	0.007
IF/TA score, mean ± SD	1.23 ± 0.83	1.07 ± 0.96	1.35 ± 0.70	0.11
cv score, mean ± SD	1.37 ± 0.83	1.11 ± 0.69	1.56 ± 0.88	0.03
C4d score, mean ± SD	1.25 ± 1.35	0.46 ± 1.0	1.8 ± 1.29	<0.001
TCMR prior to DSA occurrence, *n* (%)	13 (18.8)	6 (20.7)	7 (17.5)	0.76
**Type of ABMR**				
Active/chronic active ABMR, *n* (%)	25 (62.5)/15 (37.5)	-	25 (62.5)/15 (37.5)	-
Pure ABMR/mixed rejection, *n* (%)	29 (72.5)/11 (27.5)	-	29 (72.5)/11 (27.5)	-

Multiple DSAs were detected in 15 patients with a mean DSA number of 1.31. DSA against class I HLA were detected in 23.2% of patients, DSA against class II HLA in 69.6% of patients, and against both class I and class II HLA in 7.2% of patients. The iDSA belonged to class I in 26.1% of patients and to class II in 73.9% of patients. These distributions were not significantly different between the DSA+/ABMR+ and DSA+/ABMR– groups (Table 1). Conversely, the median iDSA MFI value was higher in the DSA+/ABMR+ than in the DSA+/ABMR– group: 7,700 [4,325–20,000] vs. 5,460 [1,975–8,650], *p* = 0.05. Graft loss occurred in 13 patients (18.8%), and no patient died during the follow-up.

In the DSA+/ABMR+ group, 38 patients (95%) had at least five plasma exchange sessions followed by four infusions of high-dose IVIg (2 g/kg). Twelve patients (30%) received one perfusion of rituximab (375 mg/kg). Two patients did not receive any specific ABMR treatment, one because of severe graft function impairment and the other one because of active tuberculosis at the time of ABMR diagnosis. In the DSA+/ABMR– group, no patient received any specific treatment.

### DSA Are Composed of all IgG Subclasses With a Specific Distribution

**Figure 2:**
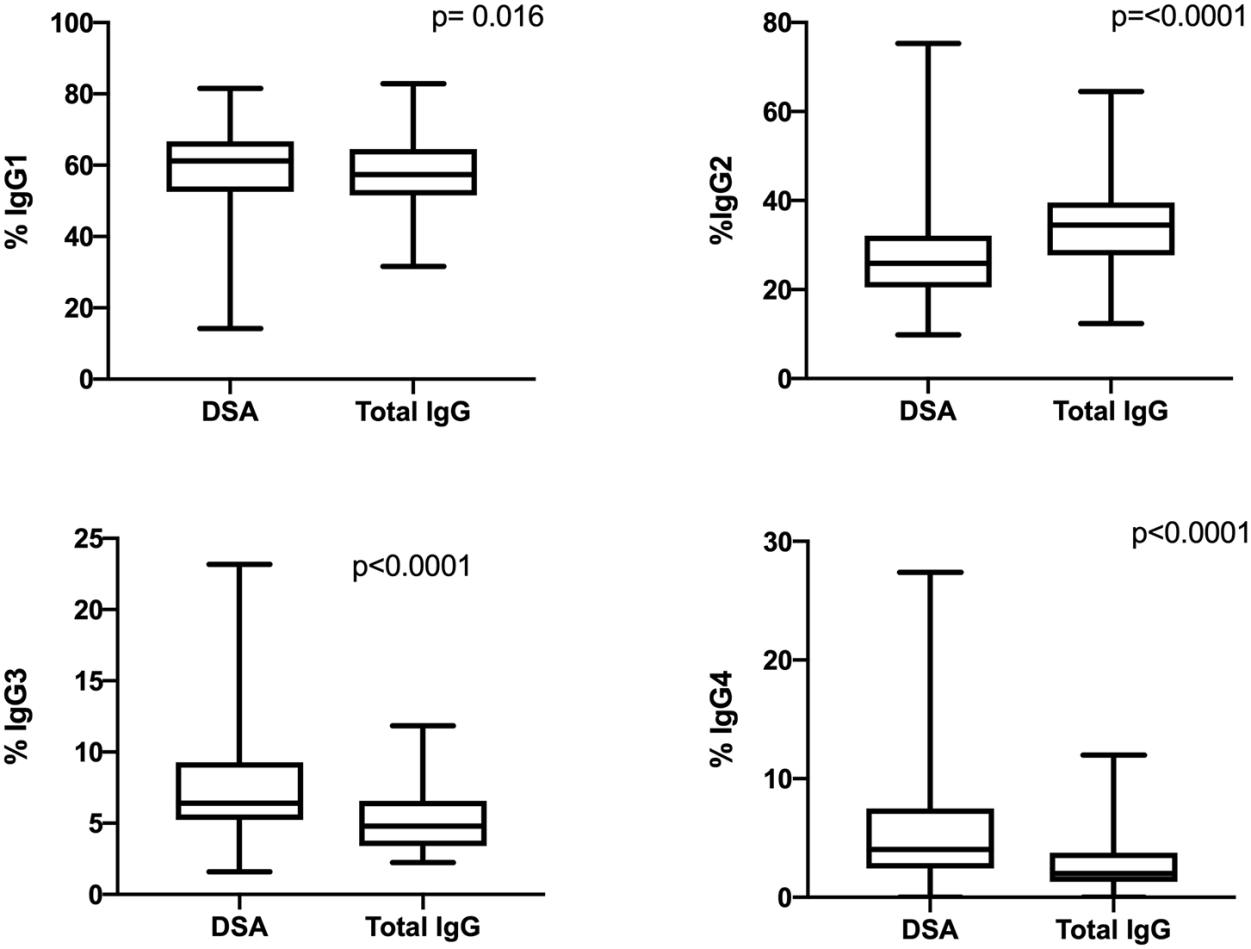
Comparison of the IgG subclass distribution in total IgG and iDSA samples from all patients.

The IgG subclass distribution was analyzed in each iDSA and total IgG sample by mass spectrometry. The four IgG subclasses were detected in all iDSA and total IgG samples (Figure 2) but with different distributions. The proportion of IgG1 (62.7 vs. 58.2%, *p* = 0.016), IgG3 (6.6 vs. 4.9%, *p* < 0.0001), and IgG4 (4.2 vs. 2.0%, *p* < 0.0001) was higher, whereas that of IgG2 was lower (26.6 vs. 35.0%, *p* < 0.0001) in iDSA than in total IgG samples (Figure 2).

### Percentage of IgG3 iDSA Is Correlated With ABMR Occurrence and Severity

**Figure 3:**
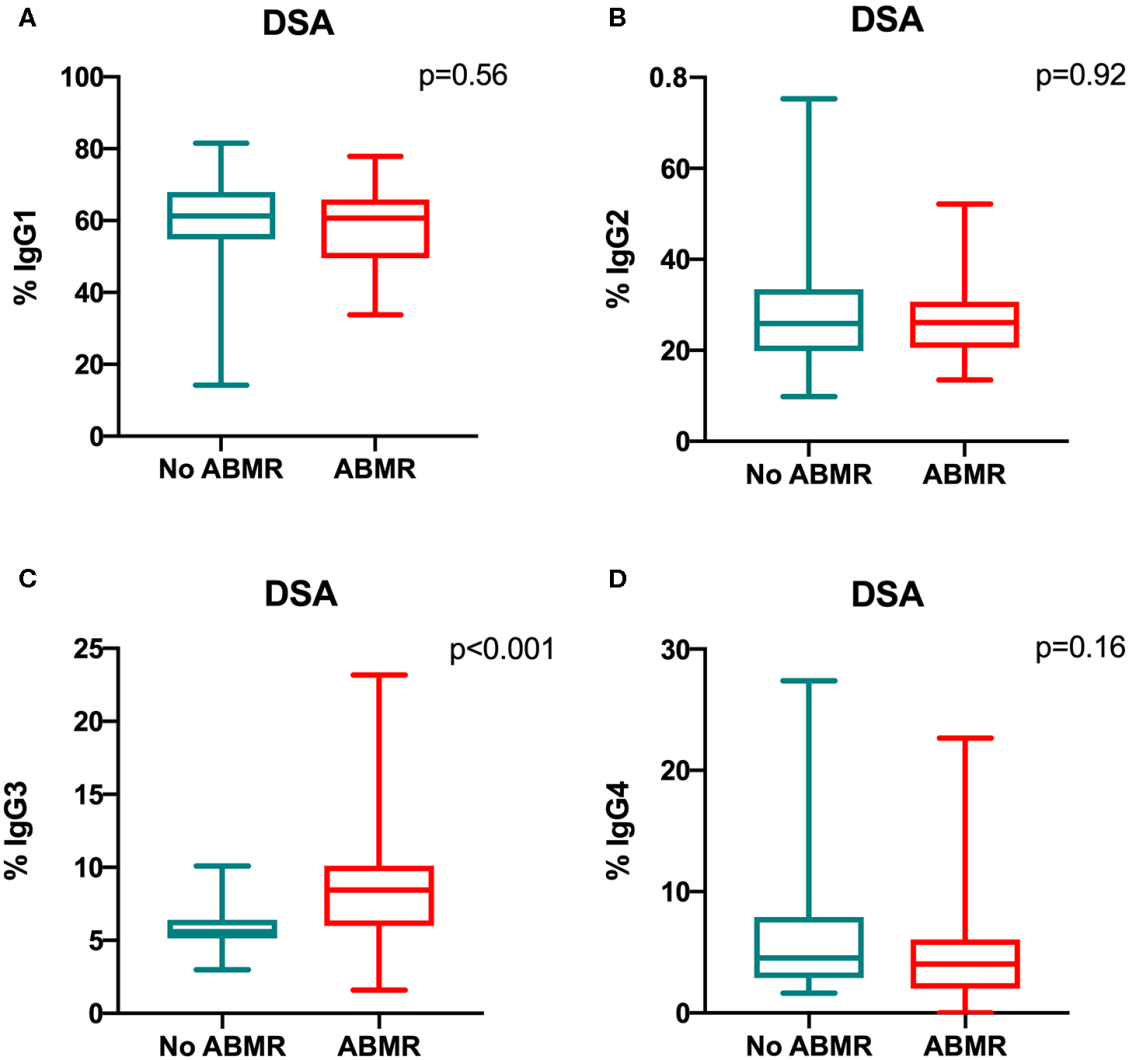
Comparison of the IgG subclass distribution in *de novo* iDSA isolated from DSA+/ABMR+ and DSA+/ABMR– patients. Proportion of IgG1 **(A)**, IgG2 **(B)**, IgG3 **(C)** and IgG4 **(D)** which composed iDSA in ABMR+ and ABMR− groups.

The percentage of IgG3 iDSA was significantly higher in the DSA+/ABMR+ compared to DSA+/ABMR– group: 8.4% [5.9–10.1] vs. 5.6% [5.1–6.4], respectively, *p* < 0.001 (Figure 3C). Conversely, the percentage of IgG1, IgG2, and IgG4 iDSA was comparable between groups (Figures 3A,B,D).

**Figure 4:**
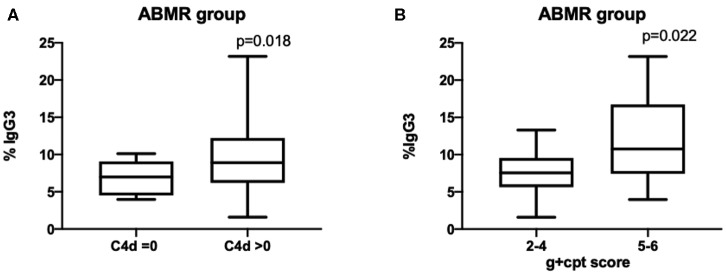
Percentage of IgG3 DSA is correlated with C4d deposition and microvascular inflammation scores.

Moreover, the IgG3 iDSA percentage was significantly associated with ABMR severity. In the group DSA+/ABMR+, IgG3 iDSA level was higher when C4d was positive (8.9% [6.2–12.2] vs. 7.0% [4.5–9.1]) than when C4d was negative, *p* = 0.018 (Figure 4A). IgG3 level was also associated with the intensity of microvascular inflammation: 10.8% [7.4–16.7] in patients with g+ptc>4 vs. 7.6% [5.6–9.6] in patients with g+ptc ≤ 4, *p* = 0.022 (Figure 4B).

Univariate and multivariate logistic regression analyses to identify patient and DSA characteristics associated with ABMR diagnosis (Table 2) indicated that high IgG3 iDSA level and proteinuria/creatininuria ratio were independent risk factors of ABMR (odds ratio [OR] = 11.15, 95% CI [2.24–55.37], *p* = 0.003, and OR = 5.18, 95% CI [1.14–23.48], *p* = 0.033, respectively).

**Table 2:** Patients and DSA characteristics associated with ABMR.

	**Univariate analysis**				**Multivariate analysis**			
**Variables**	**OR**	**95% CI**		***p*-value**	**OR**	**95% CI**		***p*-value**
Recipient sex (male)	0.86	0.32	2.26	0.765				
Recipient age (years)	1.00	0.97	1.03	0.831				
Transplantation rank	2.16	0.52	9.00	0.287				
Donor age (years)	0.97	0.95	1.00	0.160				
Donor type (living vs. deceased)	1.44	0.328	6.30	0.628				
Cold ischemia time (h)	3.16	1.16	8.59	0.024	1.10	0.99	1.22	0.051
Delay graft function	0.46	0.07	3.00	0.421				
HLA MM	0.87	0.62	1.22	0.420				
iDSA class (II vs. I)	0.83	0.27	2.51	0.754				
DSA number	1.60	0.48	5.31	0.443				
iDSA MFI (log)	3.59	1.09	11.78	0.035				
DSA Sum MFI (log)	3.53	1.13	10.96	0.029				
DSA IgG3 (high vs. Low)^a^	6.12	2.12	17.65	0.001	11.15	2.24	55.37	0.003
ATG induction	0.69	0.23	2.04	0.505				
Corticosteroids	0.74	0.25	2.20	0.591				
Tacrolimus	0.21	0.07	0.63	0.006				
Cyclosporine	12.00	1.46	98.60	0.021				
mTOR inhibitors	1.01	0.28	3.59	0.978				
IMPDH inhibitors	1.03	0.21	5.03	0.963				
GFR (ml/min/1.73 m^2^)	0.99	0.97	1.01	0.519				
Proteinuria/creatininuria (log g/g)	2.19	0.86	5.60	0.099	5.18	1.14	23.48	0.033

a *Low and high IgG3 DSA were defined according to the percentage of IgG3: <6.5 and >6.5%, respectively*.

### Higher IgG3 iDSA Percentage Is Associated With Poorer Outcome

Patients were then divided into two sub-groups according to the IgG3 iDSA percentage: “Low IgG3” (patients with IgG3 <6.4%) and “High IgG3” (patients with IgG3>6.4%) and according to the iDSA MFI value: “Low MFI group” (patients with MFI <7,500) and “High MFI” (patients with MFI > 7,500). The cutoff values of 6.4% for IgG3 and 7,500 for MFI were determined based on the ROC curve analysis and corresponded to the most relevant values for ABMR prediction (Supplementary Figure 1). IgG3 percentage has shown a better AUC than MFI although not statistically significant (0.76 [0.61–0.86] vs. 0.62 [0.51–0.77], respectively, *p* = 0.3). Sensitivity and specificity were 72.5 and 72.4% for IgG3 and 62.5 and 62.1% for MFI, respectively.

Comparison of the characteristics of the Low IgG3 and High IgG3 groups (Table 3) showed higher g+ptc score (3.2 vs. 1.8, *p* = 0.006), cv score (1.8 vs. 1.0, *p* < 0.0001), C4d score (1.6 vs. 0.9, *p* = 0.048), and proteinuria/creatininuria ratio (0.41 vs. 0.25g/g, *p* = 0.05) in the High IgG3 compared with the Low IgG3 group. Conversely, iDSA class distribution, iDSA MFI value, and eGFR were similar between groups.

**Table 3:** Characteristics of the low and high IgG3 groups and low and high MFI groups at biopsy time.

	**Patients with low IgG3 DSA (*n* = 35)**	**Patients with high IgG3 DSA (*n* = 34)**	***p*-value**	**Patients with low MFI (*n* = 39)**	**Patients with high MFI (*n* = 30)**	***p*-value**
**DSA characteristics**						
iDSA Class			0.99			0.002
*iDSA class I*	9 (25.7)	9 (26.5)		16 (41)	2 (6.7)	
*iDSA class II*	26 (74,3)	25 (73,5)		24 (59)	28 (93.3)	
MFI DSA	6,000 [2,000–12,000]	7,100 [4,225–19,380]	0.39	4,000 [1,900–5,500]	19,585 [10,770–21,360]	<0.0001
**Histological characteristics**						
g + cpt score, mean ± SD	1.8 ± 1,7	3.2 ± 2.0	0.006	1.8 ± 1,5	3.3 ± 1.8	0.003
i + t score, mean ± SD	1.3 ± 1.6	1.5 ± 1.7	0.56	1.1 ± 1.5	1.8 ± 1.8	0.06
v score, mean ± SD	0.1 ± 0.5	0.1 ± 0.2	0.99	0.1 ± 0.2	0.1 ± 0.6	0.81
cg score, mean ± SD	0.2 ± 0.5	0.5 ± 0.9	0.08	0.1 ± 0.5	0.6 ± 0.8	0.002
IF/TA score, mean ± SD	1.3 ± 0.9	1.2 ± 0.8	0.85	1.1 ± 0.8	1.3 ± 0.8	0.29
cv score, mean ± SD	1.0 ± 0.6	1.8 ± 0.9	<0.001	1.4 ± 0.8	1.3 ± 0.8	0.38
C4d score, mean ± SD	0.9 ± 1.3	1.6 ± 1.4	0.048	0.9 ± 1.2	1.8 ± 1.3	0.01
**Clinical characteristics**						
Serum creatinine (μmol/L)	127 [107–178]	151 [120–200]	0.06	149 [114–203]	145 [113–185]	0.55
eGFR MDRD (ml/min/1.73 m^2^)	47 [38–64]	44.5 [24–51.3]	0.09	44 [28–56]	48 [34–60]	0.41
PU/CU ratio (g/g)	0.25 [0.10–0.42]	0.41 [0.16–1.55]	0.05	0.33 [0.16–0.90]	0.21 [0.10–0.72]	0.20
Time post Tx (years)	4.1 [1.0–7.1]	7.7 [3.0–10.3]	0.02	3.7 [0.9–7.9]	6.9 [4.1–11.2]	0.003
ABMR status			<0.001			0.09
No ABMR, *n* (%)	25 (65.7)	6 (17.6)		20 (51.3)	9 (30)	
ABMR rejection, *n* (%)	12 (34.3)	28 (82.4)		19 (48.7)	21 (70)	
Subclinical ABMR, *n* (%)	6 (17.1)	10 (29.4)		9 (23.1)	7 (23.3)	
Clinical ABMR, *n* (%)	6 (17.1)	18 (52.9)		10 (25.6)	14 (46.7)	

*PU, proteinuria*.

*CU, creatininuria*.

*Time post Tx, time post-transplantation*.

Comparison of the characteristics of the Low and High MFI groups (Table 3) showed that the percentage of class II iDSA, g+ptc score (3.3 vs. 1.8, *p* = 0.003), cg score (0.6 vs. 0.1, *p* = 0.002), and C4d score (1.8 vs. 0.9, *p* = 0.01) were higher in the High than in the Low MFI group.

**Figure 5:**
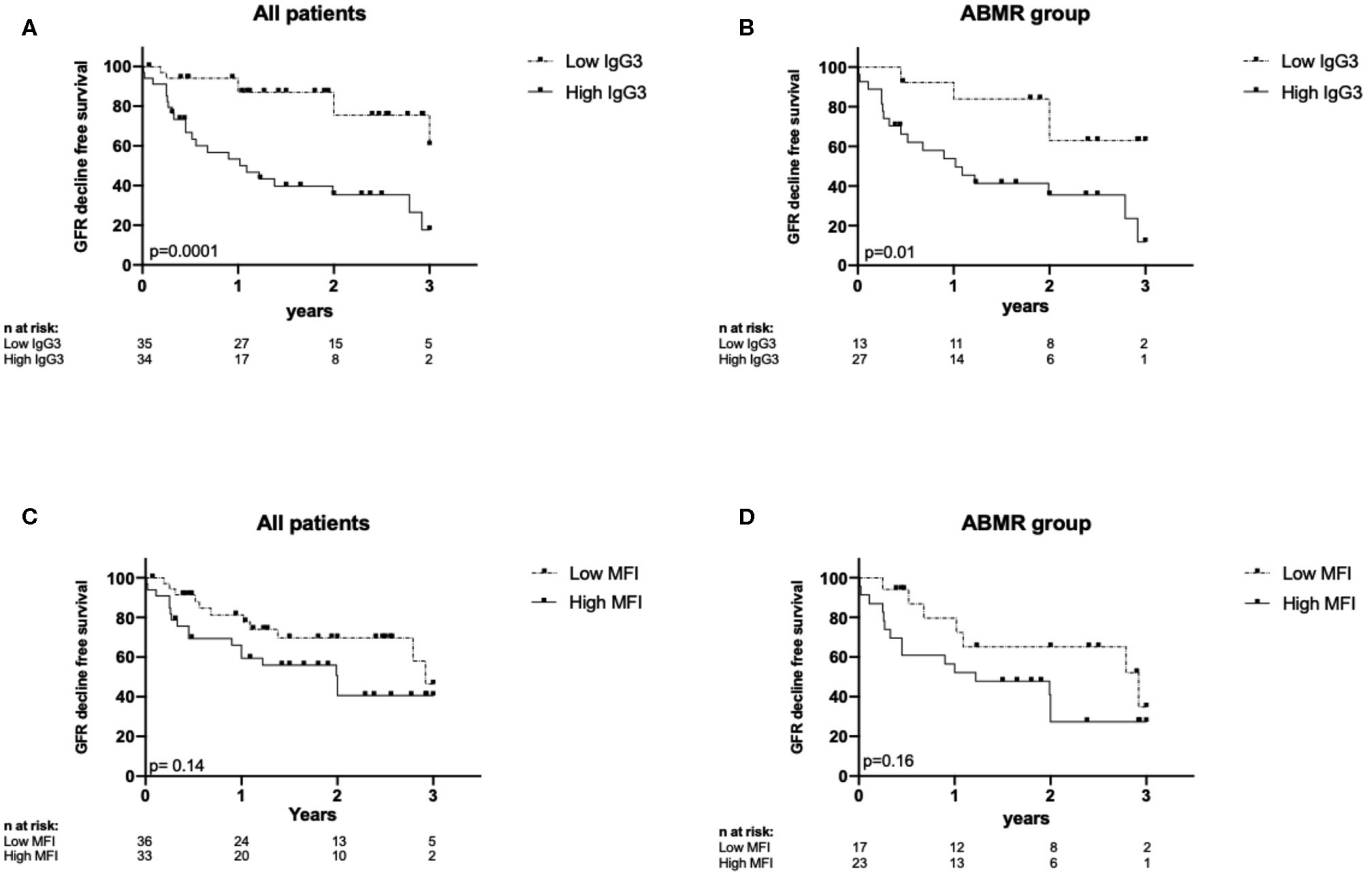
Kaplan-Meyer curves for eGFR decline-free survival according to IgG3 level in all patients **(A)** and in DSA+ABMR+ group **(B)**, and according to MFI level in all patients **(C)** and in DSA+ABMR+ group **(D)**.

The eGFR decline-free survival rate was lower in the High than in the Low IgG3 group (Figure 5A, *p* = 0.001). At 1 year post-graft, eGFR decrease >25% was observed in 50% of patients in the High IgG3 group and in 11.4% of patients in the Low IgG3 group. The same analysis only in DSA+/ABMR+ patients showed again that high IgG3 iDSA was associated with higher risk of impaired renal function (Figure 5B, *p* = 0.01). Conversely, the eGFR decline-free survival rate was not significantly different in the Low and High MFI groups from the entire sample (Figure 5C) and from the DSA+/ABMR+ group (Figure 5D). Percentage of IgG1, IgG2, and IgG4 did not affect either eGFR decline >25%.

Graft survival was not significantly different in the Low IgG3 and High IgG3 DSA groups and in the Low MFI and High MFI groups (data not shown). Finally, univariate and multivariate Cox regression analyses (Table 4) showed that high IgG3 iDSA (HR = 5.68, 95% CI [1.82–17.8], *p* = 0.003), low eGFR at time of biopsy (HR = 0.95, 95% CI [0.93–0.98], *p* = 0.002), and number of DSA (HR = 2.04, 95% CI [1.17–3.58], *p* = 0.013) were independent risk factors of eGFR decline >25%.

**Table 4:** Patients and DSA characteristics associated with GFR decline.

	**Univariate analysis**				**Multivariate analysis**			
**Variables**	**HR**	**95% CI**		***p*-value**	**HR**	**95% CI**		***p*-value**
Recipient sex (male)	0.48	0.23	1.03	0.059				
Recipient age (years)	0.99	0.96	1.01	0.287				
Donor age (years)	0.97	0.95	0.99	0.017				
Donor type (living vs. deceased)	1.75	0.41	7.4	0.448				
Cold ischemia time (h)	1.05	0.50	2.21	0.890				
HLA mismatches	0.90	0.68	1.19	0.448				
iDSA class (II vs. I)	0.73	0.33	1.60	0.432				
DSA number	1.75	1.16	2.65	0.008	2.04	1.17	3.58	0.013
iDSA MFI (log)	2.18	0.91	5.2	0.079				
DSA IgG3 (high vs. Low)	4.23	1.80	9.97	0.001	5.69	1.82	17.8	0.003
ATG induction	0.47	0.22	0.98	0.045				
Corticosteroids	0.84	0.37	1.91	0.684				
Tacrolimus	0.60	0.29	1.25	0.170				
Cyclosporine	1.08	0.44	2.67	0.871				
mTOR inhibitors	0.79	0.30	2.09	0.641				
IMPDH inhibitors	1.60	0.38	6.77	0.521				
GFR (ml/min/1.73 m^2^)	0.96	0.94	0.98	0.001	0.95	0.93	0.98	0.002
PU/CU (log g/g)	1.12	0.96	1.30	0.157				
C4d score	1.77	1.30	2.40	0.001				
g + cpt score	1.17	0.96	1.43	0.126				
v score	0.77	0.26	2.23	0.625				
i + t score	1.14	0.91	1.41	0.276				
cg score	1.54	0.99	2.39	0.054				
FIAT score	1.67	1.07	2.61	0.025				

*PU, proteinuria*.

*CU, creatininuria*.

*Time post Tx, time post-transplantation*.

## Discussion

Here, we assessed the distribution of the donor IgG subclasses by mass spectrometry after iDSA occurrence. We found for the first time that all *de novo* DSA were composed of all IgG subclasses and that higher percentage of IgG3 iDSA was correlated with ABMR occurrence and severity, and poorer graft outcome.

By using mass spectrometry after iDSA isolation, we detected the four IgG subclasses in each isolated iDSA. We demonstrated that the alloimmune response is always polyclonal. Contrary to Luminex technique, this innovative method not only detected the different IgG subclass, but also allowed their relative quantification. We found that IgG1 is the predominant DSA subclass (62.2% of the total IgG DSA), followed by IgG2 (23.1%), IgG3 (7.8%), and IgG4 (4.7%). Previous studies that analyzed the composition of DSA Ig subclasses and their impact on kidney transplantation outcome adapted the generic Luminex Single Antigen assay for DSA subclass detection by flow cytometry (9, 10, 19–21). They used secondary antibodies specific for the different IgG subclasses detection, and defined the presence of a subclass by an MFI value above a fixed cutoff. In this way, Lefaucheur et al. detected a single subclass in 31% of iDSA (mainly IgG1), two and three subclasses in 21% and 24% of iDSA, respectively, and the four subclasses only in 7% of iDSA (10). By using the Luminex IgG assay, any subclass was detected in 16% to 21% of iDSA, especially in iDSA with low MFI value (10, 20, 21). This suggests that this test lacks sensitivity for subclass detection. Moreover, cross-reactivity of secondary antibodies could be observed when they are not fully specific for one subclass. However, this non-specific reactivity seems to have not exceeded 5% of the binding (22).

Our study showed that the DSA class composition is antigen-specific and that IgG subclass distribution is different from that in total IgG. Indeed, the IgG1, IgG3, and IgG4 fractions were higher in the DSA antibody population than in total IgG. Immunoglobulin class switching is a complex mechanism that mainly depends on the antigen nature and the interaction between B cells and their environment. Polysaccharide and glycolipid antigens usually promote IgG2 production (23) and protein antigens mainly drive IgG1 and IgG3 production (24). On the other hand, chronic antigen exposure leads to IgG4 production (25). The B-cell microenvironment is also involved in class switching. Class switching typically occurs following interaction with CD4+ T cells via the receptors CD40–CD40L and cytokines (26, 27). Avery et al. elegantly showed that IL-4 induces the switch to IgG1 in CD40L-stimulated human naive B cells, whereas IL-21 mainly induces IgG3 production (28). High percentage of IgG1 and IgG3 could easily be explained by a T-dependent alloantigen presentation after kidney transplantation. Moreover, high IgG4 percentage is not surprising in a context of chronic antigen exposure, as observed in patients who underwent kidney graft. However, the signals that control isotype switching are incompletely defined, and more studies are needed, particularly in the context of alloimmunization to improve ABMR understanding.

Several studies reported that detection of IgG3-iDSA with the Luminex test is associated with higher ABMR risk after transplantation of kidney and also of other organs, particularly liver (10, 20, 29–31). Lefaucheur et al. detected IgG3 in 23% of iDSA, and their presence was associated with ABMR and poorer graft outcome (10). Our results confirmed IgG3's deleterious role and also highlighted the predominant role of IgG3 relative proportion in ABMR risk. Indeed, our technique allowed comparing the relative proportion of each iDSA subclass and showing that ABMR was associated with iDSA IgG3 > 6.4%, independently of the other subclass distribution.

IgG1 and IgG3 are known to activate the classical complement pathway, and IgG3 display the best ability to bind to and activate the complement cascade (7). In agreement, the percentage of IgG3 iDSA was associated with C4d-positive ABMR and with eGFR decline in our study. Conversely, IgG1 percentage was similar in DSA+/ABMR+ and DSA+/ABMR– patients. Moreover, the IgG subclass affects also the affinity for FcγRIIIa and the capacity for natural killer cell recruitment (8). IgG3 is the subclass with the most affinity for this receptor, and we found a correlation between the inflammatory microvascular lesions and IgG3 percentage.

Finally, we found that high MFI was a risk factor for ABMR, as previously reported (5, 32–34). However, MFI value was not correlated with the IgG3 level. In our multivariate analysis, the only three factors associated with GFR decline were eGFR at diagnosis, number of DSA, and high IgG3 percentage. While high IgG3 percentage was associated with ABMR occurrence and histological severity and also with eGFR decline, the difference in graft survival was not statistically significant between the High and Low IgG3 groups. This is explained by limited number of graft losses (18.8%) in a relatively short follow-up period (mean: 1.7 years). In previous studies, the median time to graft loss after ABMR diagnosis was >5 years (35).

Our work has some limitations. First, we did not monitor the iDSA subclass distribution at different times during the follow-up, but only at the time of kidney biopsy after DSA occurrence. Therefore, we do not know whether the iDSA subclass proportions vary over time, and whether changes are influenced by ABMR treatment. Second, we focused only on *de novo* DSA. As the therapeutic management and clinical course after pre-formed DSA and *de novo* DSA are not similar (36), our results cannot be extrapolated to patients with pre-formed DSA. Moreover, we did not analyze iDSA with the C1q or C3d Luminex assays, which would have allowed comparing the percentage of IgG3 in mass spectrometry and DSA ability to fix complement *in vitro*. However, the routine use of C1q/C3d Luminex assays to better predict rejection and graft loss remains controversial (37, 38). Finally, our mass spectrometry-based technique is relatively complex, although it brings useful information to understand ABMR mechanism.

In conclusion, we reported for the first time that *de novo* DSA are polyclonal antibodies always composed of the four IgG subclasses, and that the IgG3 proportion is crucial for their pathogenicity. High IgG3 percentage is associated with ABMR occurrence and severity and with higher risk of eGFR decline. Better knowledge of the mechanism implicated in class switching of DSA may lead to the development of new therapeutic approaches in ABMR.

## Data Availability Statement

The datasets generated for this study are available on request to the corresponding author.

## Ethics Statement

The studies involving human participants were reviewed and approved by CPP Ile de France V. The patients/participants provided their written informed consent to participate in this study.

## Author Contributions

VP and ML designed the study, analyzed the data, and wrote the paper. IS performed the statistical analyses. CL, AB, and NB reviewed the paper. VP, NB, CS, and EP-G performed the experiments. NB and CL participated in data analysis. All authors had reviewed the paper and provided final approval of the version to be published.

## Conflict of Interest

The authors declare that the research was conducted in the absence of any commercial or financial relationships that could be construed as a potential conflict of interest.

## References

[1] Sellarés J de Freitas DG Mengel M Reeve J Einecke G Sis B. Understanding the causes of kidney transplant failure: the dominant role of antibody-mediated rejection and nonadherence. Am J Transplant. (2012) 12:388–99. 10.1111/j.1600-6143.2011.03840.x 22081892

[2] Stegall MD Chedid MF Cornell LD. The role of complement in antibody-mediated rejection in kidney transplantation. Nat Rev Nephrol. (2012) 8:670–8. 10.1038/nrneph.2012.212 23026942

[3] Everly MJ Rebellato LM Haisch CE Ozawa M Parker K Briley KP. Incidence and impact of de novo donor-specific alloantibody in primary renal allografts. Transplantation. (2013) 95:410–7. 10.1097/TP.0b013e31827d62e3 23380861

[4] Wiebe C Gibson IW Blydt-Hansen TD Karpinski M Ho J Storsley LJ. Evolution and clinical pathologic correlations of de novo donor-specific HLA antibody post kidney transplant. Am J Transplant. (2012) 12:1157–67. 10.1111/j.1600-6143.2012.04013.x 22429309

[5] Lefaucheur C Loupy A Hill GS Andrade J Nochy D Antoine C. Preexisting donor-specific HLA antibodies predict outcome in kidney transplantation. J Am Soc Nephrol. (2010) 21:1398–406. 10.1681/ASN.2009101065 20634297 PMC2938596

[6] Loupy A Lefaucheur C Vernerey D Prugger C Duong van Huyen JP Mooney N. Complement-binding anti-HLA antibodies and kidney-allograft survival. N Engl J Med. (2013) 369:1215–26. 10.1056/NEJMoa1302506 24066742

[7] Michaelsen TE Garred P Aase A. Human IgG subclass pattern of inducing complement-mediated cytolysis depends on antigen concentration and to a lesser extent on epitope patchiness, antibody affinity and complement concentration. Eur J Immunol. (1991) 21:11–16. 10.1002/eji.1830210103 1703960

[8] Bruhns P Iannascoli B England P Mancardi DA Fernandez N Jorieux S. Specificity and affinity of human Fcgamma receptors and their polymorphic variants for human IgG subclasses. Blood. (2009) 113:3716–25. 10.1182/blood-2008-09-179754 19018092

[9] Hönger G Hopfer H Arnold ML Spriewald BM Schaub S Amico P. Pretransplant IgG subclasses of donor-specific human leukocyte antigen antibodies and development of antibody-mediated rejection. Transplantation. (2011) 92:41–7. 10.1097/TP.0b013e31821cdf0d 21637140

[10] Lefaucheur C Viglietti D Bentlejewski C Duong van Huyen JP Vernerey D Aubert O. IgG donor-specific anti-human HLA antibody subclasses and kidney allograft antibody-mediated injury. J Am Soc Nephrol. (2016) 27:293–304. 10.1681/ASN.2014111120 26293822 PMC4696574

[11] Schnaidt M Weinstock C Jurisic M Schmid-Horch B Ender A Wernet D. HLA antibody specification using single-antigen beads, a technical solution for the prozone effect. Transplantation. (2011) 92:510–5. 10.1097/TP.0b013e31822872dd 21869744

[12] Visentin J Vigata M Daburon S Contin-Bordes C Fremeaux-Bacchi V Dromer C. Deciphering complement interference in anti-human leukocyte antigen antibody detection with flow beads assays. Transplantation. (2014) 98:625–31. 10.1097/TP.0000000000000315 25058376

[13] Haas M Loupy A Lefaucheur C Roufosse C Glotz D Seron D. The banff 2017 kidney meeting report: revised diagnostic criteria for chronic active T cell-mediated rejection, antibody-mediated rejection, and prospects for integrative endpoints for next-generation clinical trials. Am J Transplant. (2018) 18:293–307. 10.1111/ajt.14625 29243394 PMC5817248

[14] Levey AS Bosch JP Lewis JB Greene T Rogers N Roth D. A more accurate method to estimate glomerular filtration rate from serum creatinine: a new prediction equation. Modification of diet in renal disease study group. Ann Intern Med. (1999) 130:461–70. 10.7326/0003-4819-130-6-199903160-00002 10075613

[15] Dowle AA Wilson J Thomas JR. Comparing the diagnostic classification accuracy of iTRAQ, peak-area, spectral-counting, and emPAI methods for relative quantification in expression proteomics. J Proteome Res. (2016) 15:3550–62. 10.1021/acs.jproteome.6b00308 27546623

[16] Ladwig PM Barnidge DR Snyder MR Katzmann JA Murray DL. Quantification of serum IgG subclasses by use of subclass-specific tryptic peptides and liquid chromatography–tandem mass spectrometry. Clin Chem. (2014) 60:1080–8. 10.1373/clinchem.2014.222208 24799527

[17] Miyamoto S Stroble CD Taylor S Hong Q Lebrilla CB Leiserowitz GS. Multiple reaction monitoring for the quantitation of serum protein glycosylation profiles: application to ovarian cancer. J Proteome Res. (2018) 17:222–33. 10.1021/acs.jproteome.7b00541 29207246 PMC6203861

[18] Yuan W Sanda M Wu J Koomen J Goldman R. Quantitative analysis of immunoglobulin subclasses and subclass specific glycosylation by LC-MS-MRM in liver disease. J Proteomics. (2015) 116:24–33. 10.1016/j.jprot.2014.12.020 25582524 PMC4329072

[19] Freitas MC Rebellato LM Ozawa M Nguyen A Sasaki N Everly M. The role of immunoglobulin-G subclasses and C1q in de novo HLA-DQ donor-specific antibody kidney transplantation outcomes. Transplantation. (2013) 95:1113–9. 10.1097/TP.0b013e3182888db6 23514959

[20] Khovanova N Daga S Shaikhina T Krishnan N Jones J Zehnder D. Subclass analysis of donor HLA-specific IgG in antibody-incompatible renal transplantation reveals a significant association of IgG4 with rejection and graft failure. Transpl Int. (2015) 28:1405–15. 10.1111/tri.12648 26264744 PMC4975692

[21] Hamdani G Goebel JW Brailey P Portwood EA Hooper DK Girnita AL. IgG3 anti-HLA donor-specific antibodies and graft function in pediatric kidney transplant recipients. Pediatr Transplant. (2018) 22:e13219. 10.1111/petr.13219 29855114

[22] Lowe D Higgins R Zehnder D Briggs DC. Significant IgG subclass heterogeneity in HLA-specific antibodies: Implications for pathogenicity, prognosis, and the rejection response. Hum Immunol. (2013) 74:666–72. 10.1016/j.humimm.2013.01.008 23369861

[23] Siber GR Schur PH Aisenberg AC Weitzman SA Schiffman G. Correlation between serum IgG-2 concentrations and the antibody response to bacterial polysaccharide antigens. N Engl J Med. (1980) 303:178–82. 10.1056/NEJM198007243030402 6966763

[24] Smith TF. IgG subclasses. Adv Pediatr. (1992) 39:101–26. 1442311

[25] Aalberse RC van der Gaag R van Leeuwen J. Serologic aspects of IgG4 antibodies. Prolonged immunization results in an IgG4-restricted response. J Immunol. (1983) 130:722–6. 6600252

[26] Aversa G Punnonen J Carballido JM Cocks BG de Vries JE. CD40 ligand-CD40 interaction in Ig isotype switching in mature and immature human B cells. Semin Immunol. (1994) 6:295–301. 10.1006/smim.1994.1038 7532459

[27] Coffman RL Lebman DA Rothman P. Mechanism and regulation of immunoglobulin isotype switching. Adv Immunol. (1993) 54:229–70. 10.1016/S0065-2776(08)60536-2 8379463

[28] Avery DT Bryant VL Ma CS de Waal Malefyt R Tangye SG. IL-21-induced isotype switching to IgG and IgA by human naive B cells is differentially regulated by IL-4. J Immunol. (2008) 181:1767–79. 10.4049/jimmunol.181.3.1767 18641314

[29] Everly MJ Rebellato LM Haisch CE Briley KP Bolin P Kendrick WT. Impact of IgM and IgG3 anti-HLA alloantibodies in primary renal allograft recipients. Transplantation. (2014) 97:494–501. 10.1097/01.TP.0000441362.11232.48 24487396

[30] Kaneku H O'Leary JG Taniguchi M Susskind BM Terasaki PI Klintmalm GB. Donor-specific human leukocyte antigen antibodies of the immunoglobulin G3 subclass are associated with chronic rejection and graft loss after liver transplantation. Liver Transplant. (2012) 18:884–892. 10.1002/lt.23451 22508525

[31] O'Leary JG Kaneku H Banuelos N Jennings LW Klintmalm GB Terasaki PI. Impact of IgG3 subclass and C1q-fixing donor-specific HLA alloantibodies on rejection and survival in liver transplantation. Am J Transplant. (2015) 15:1003–13. 10.1111/ajt.13153 25772599

[32] Schinstock CA Cosio F Cheungpasitporn W Dadhania DM Everly MJ Samaniego-Picota MD. The value of protocol biopsies to identify patients with *de novo* donor-specific antibody at high risk for allograft loss. Am J Transplant. (2017) 17:1574–84. 10.1111/ajt.14161 27977905 PMC5445019

[33] Kwon H Kim YH Choi JY Shin S Jung JH Park SK. Impact of pretransplant donor-specific antibodies on kidney allograft recipients with negative flow cytometry cross-matches. Clin Transplant. (2018) 32:e13266. 10.1111/ctr.13266 29676812

[34] de Castro MCR Barbosa EA Souza RP. The kinetics of anti-hla antibodies in the first year after kidney transplantation: in whom and when should they be monitored? J Transplant. (2018) 2018:8316860. 10.1155/2018/8316860 29850195 PMC5937436

[35] Viglietti D Loupy A Aubert O Bestard O Duong Van Huyen JP Taupin JL. Dynamic prognostic score to predict kidney allograft survival in patients with antibody-mediated rejection. J Am Soc Nephrol. (2018) 29:606–619. 10.1681/ASN.2017070749 29255058 PMC5791064

[36] Aubert O Loupy A Hidalgo L Duong van Huyen JP Higgins S Viglietti D. Antibody-mediated rejection due to preexisting versus *de novo* donor-specific antibodies in kidney allograft recipients. J Am Soc Nephrol. (2017) 28:1912–23. 10.1681/ASN.2016070797 28255002 PMC5461792

[37] Courant M Visentin J Linares G Dubois V Lepreux S Guidicelli G. The disappointing contribution of anti-human leukocyte antigen donor-specific antibodies characteristics for predicting allograft loss. Nephrol Dial Transplant. (2018) 33:1853–63. 10.1093/ndt/gfy088 29672702

[38] Yell M Muth BL Kaufman DB Djamali A Ellis TM. C1q binding activity of *de novo* donor-specific hla antibodies in renal transplant recipients with and without antibody-mediated rejection. Transplantation. (2015) 99:1151–5. 10.1097/TP.0000000000000699 25839705

