# Supplementary material for: Distribution of de novo Donor-Specific Antibody Subclasses Quantified by Mass Spectrometry: High IgG3 Proportion Is Associated With Antibody-Mediated Rejection Occurrence and Severity
Source: Front Immunol. 2020 Jun 2;11:919. doi: 10.3389/fimmu.2020.00919 (PMC7326073; doi:10.3389/fimmu.2020.00919)

SUPPLEMENTARY TABLE AND FIGURE:

Table S1 : IgG subclass-specific proteotypic sequences used for the subclass

Uniprot accession number	Human IgG subclass	Unique proteotypic sequences
P01857	IgG1 chain C region	GPSVFPLAPSSK, TTPPVLDSDGSFFLYSK
P01859	IgG2 chain C region	GLPAPIEK, VVSVLTVVHQDWLNGK
P01860	IgG3 chain C region	SCDTPPPCPR, TPLGDTTHTCPR
P01861	IgG4 chain C region	EPQVYTLPPSQEEMTK, TTPPVLDSDGSFFLYSR

Figure S1 : Receiver operating characteristics (ROC) curves to evaluate the discriminative power of MFI and IgG3 proportion for the diagnosis of acute antibody-mediated rejection

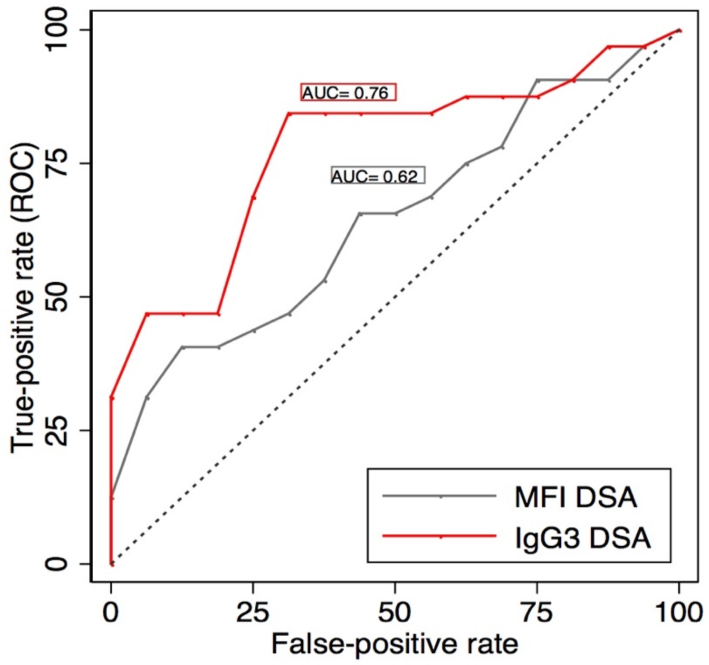

Supplement: Supplementary file 1 [file Data_Sheet_1.PDF]
